# Analysis of the Aerosol Generated from Tetrahydrocannabinol, Vitamin E Acetate, and Their Mixtures

**DOI:** 10.3390/toxics10020088

**Published:** 2022-02-15

**Authors:** Vladimir B. Mikheev, Alexander Ivanov

**Affiliations:** Battelle Memorial Institute, 505 King Avenue, Columbus, OH 43201, USA; ivanova@battelle.org

**Keywords:** e-cigarette, vaping, product use–associated lung injury (EVALI), tetrahydrocannabinol (THC), vitamin E acetate (VEA), thermal degradation, particle size distribution

## Abstract

E-cigarette, or vaping, product use–associated lung injury (EVALI) outbreak was linked to vitamin E acetate (VEA) used as a solvent for tetrahydrocannabinol (THC). Several studies were conducted to assess the products of VEA (and THC/VEA mixtures) thermal degradation as a result of vaporizing/aerosolizing from a traditional type (coil—cotton wick) and ceramic type coil vape pens. The particle size distribution (PSD) of VEA aerosol and the temperature VEA and THC/VEA mixtures are heated to were also measured for a few types of traditional and ceramic vape pens. The current study assessed the PSD of the aerosol generated from THC, VEA, and a number of THC/VEA mixtures using a dab-type vape pen under two different temperature settings and two puffing flow rates. Thermal degradation of THC, VEA, and THC/VEA mixtures were also assessed, and coil temperature was measured. Results showed the dependence of the PSD upon the chemical content of the aerosolized mixture as well as upon the puffing flow rate. Minimal thermal degradation was observed. Flaws in the vape pen’s design, which most likely affected results, were detected. The suitability of VEA, THC, and THC/VEA mixtures with certain types of vape pens was discussed.

## 1. Introduction

The emergence of an e-cigarette, or vaping, product use–associated lung injury (EVALI) crisis [1] raised a number of questions, but scientists still did not obtain satisfactory answers to explain the nature of this outbreak [2]. A correlation was observed between the presence of vitamin E acetate (VEA) in the lungs and EVALI appearance [3,4]. VEA was often used as a solvent with tetrahydrocannabinol (THC) containing e-liquids [5]; therefore, several studies were devoted to investigating which toxic chemicals could be created as a result of VEA heating followed by aerosolizing [6,7,8,9,10,11]. A number of potential toxicants were detected such as ketene, 1-pristene, duroquinone (DQ), durohydroquinone (DQH), durohydroquinone monoacetate (DHQMA), and 2,6,10,14-tetramethyl-1-pentadecene (TMPD) [6,7,8,9,11,12], and high heating temperatures (above 500 °C) were observed while vaporizing VEA containing e-liquids from commercially available vape pens [8,9]. Beyond chemical toxicity resulting from the inhalation of products of VEA pyrolysis, possible VEA impact on pulmonary injury [13] and the mechanical properties of pulmonary lung surfactants were also discussed [14].

Another critically important aspect of aerosol toxicity is particle size since it defines which region of the human respiratory system will be most affected by the inhaled aerosol. Two studies were performed on the measurements of the particle size distribution of VEA containing aerosols [9,15]. In our earlier study, a differential mobility spectrometer was applied to measure the count median diameter (CMD) of the aerosol generated out of pure VEA using four different types of vape pens [9] (one was a so-called dab-type pen designed for wax-type mixtures containing a high concentration of THC, two other vape pens were designed for liquid oil-type cannabinoid mixtures, and the last one was designed for traditional propylene glycol and glycerol containing mixtures). Heating power and puffing flow rate were varied, and CMD was observed within the 50–200 nm range. In addition, mass median aerodynamic diameter (MMAD) was measured using an inertial impactor for the traditional type vape pen listed above, and MMAD varied from 308 nm to 370 nm. Temperature measurements of the heating coil and non-targeted chemical analysis of the aerosol generated by the same type of vape pen were also conducted. Measured temperatures reached 500–600 °C, and 1-pristene, DQ, and DHQMA were tentatively identified as the products of VEA thermal decomposition.

An independent study conducted by another group of researchers [15] measured the MMAD of the aerosol generated out of various THC diluents (including VEA) with a commercially available (NJOY top tank) vape pen (designed for propylene glycol and glycerol mixtures) using an inertial impactor technique and obtained a 610 nm average MMAD for VEA aerosol. Heating temperature measurements for a 50%/50% THC/VEA mixture vaped using a ceramic type vertically positioned coil vape pen along with chemical analysis of VEA aerosol was conducted by another independent group [8] and showed temperatures ranging from 375–569 °C as well as a number of VEA thermal degradation products such as 1-pristine, DQ, DHQMA, and a compound consistent with 4-acetoxy- 2,3,5-trimethyl-6-methylene-2,4-cyclohexadienone.

Summarizing the current state of VEA and THC aerosol physical and chemical characterization conducted under controlled heating temperatures: no PSD measurements were performed on the aerosol generated from THC or THC/VEA mixtures using a vape pen designed to aerosolize cannabinoid products. Heating temperature measurements along with the chemical analysis of the THC and VEA aerosol were conducted using two types of vape pens for a limited set of chemical mixtures (VEA and 50%/50% THC/VEA).

The goal of the present study was to conduct PSD and heating temperature measurements along with a preliminary chemical evaluation of the aerosol generated out of VEA, THC, and THC/VEA mixtures of various proportions using a vape pen designed for aerosolizing cannabinoid products.

## 2. Materials and Methods

### 2.1. Vape Pen

**Figure 1:**
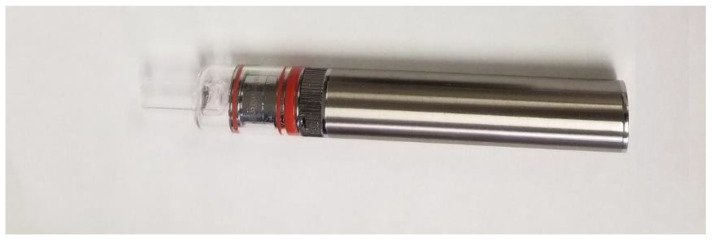
Kind Dream vape pen.

Four vape pens were used in the previous study: Kind Discreet (ceramic type horizontally positioned coil), Kind Mist (ceramic type vertically positioned coil), Kind Dream (dab-type ceramic with two horizontally positioned coils), and Joyetech eVic (horizontal coil wrapped around a cotton wick) [9]. The Kind Dream (dab-type) device (Figure 1), which belongs to one of the fastest-growing groups in the cannabis market [16], was chosen for our study because it allowed us to test the entire range of THC/VEA mixtures: VEA, 20%/80% THC/VEA, 50%/50% THC/VEA, 80%/20% THC/VEA, and THC. VEA, as well as THC/VEA mixtures, are liquid oils that can be loaded into basically any type of vape pen. THC is a highly viscous wax, so it was extremely difficult to load it into the vape pen tank designed for liquids and then clean the tank after the test. 

### 2.2. Chemicals

VEA (≥96% HPLC grade) was purchased from MilliporeSigma, Burlington, Massachusetts, US. THC (85.22% of d9-THC, 95.12% of total cannabinoids, HPLC) was legally imported according to Drug Enforcement Administration (DEA) regulations from one of the Battelle’s clients. Fresh mixtures of THC/VEA (20%/80% or 50%/50% or 80%/20% measured by weight) were prepared every day prior to testing. A total of 1.5 mL of chemicals was loaded into the vape pen prior to each test (and cleaned/sonicated with methanol after each test). Unused THC containing mixtures were disposed of at the end of the working day. 

In addition, the following chemicals were used for chemical analysis: methylene chloride (≥99.9% purity; MilliporeSigma; Burlington, MA, USA) and a stock solution of six isotopically labeled internal standards (acenaphthene-d10, chrysene-d12, 1,4-dichlorobenzne-d4, naphthalene-d8, perylene-d12, and phenanthrene-d10, each with a purity of ≥98% and at a concentration of 4 mg/mL) in methylene chloride (Restek^®^; Bellefonte, PA, USA).

### 2.3. Temperature Measurements

**Figure 2:**
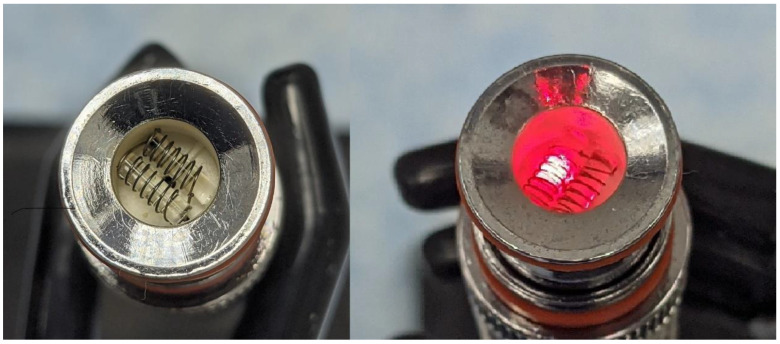
Kind Dream heating element (**left**—coils, **right**—heated coils).

The Kind Dream vape pen allows for three heating temperature set points of low, medium, and high (350 °F, 390 °F, and 430 °F, respectively). Two temperature settings were used during the tests: low 350 °F (~177 °C) and high 430 °F (~221 °C). An infra-red IR-thermometer (Micro Epsilon, Model-CTLM-3H1-CF3-C3, Ortenburg, Germany) that allows temperature monitoring in real-time (every 0.1 s) was used for independent temperature measurements. To conduct temperature measurements, the glass mouthpiece of the vape pen was removed (Figure 2), chemicals (VEA, or 50%/50% THC/VEA, or THC) were loaded into the vape pen, and the infra-red beam was directed onto the heating coil. The temperature of the heating coil was monitored for each chemical mixture at both temperature set points while heating power was turned on for 5 s then off for 60 s. This cycle was repeated eight times to simulate puffing topography conditions used for aerosol generation. Each set of measurements consisted of 8 “puffs” and was repeated at least three times.

### 2.4. Aerosol Size Distribution Measurements

The Differential Mobility Spectrometer (DMS500) along with the Smoking Cycle Simulator (both manufactured by Cambustion Ltd., Cambridge, UK) were used for real-time particle size characterization: eight 5 s puffs (60 s inter-puff interval) per test with three replicates per set of the test conditions. VEA, 20%THC-80%VEA, 50%VEA-50%THC, 80%THC-20%VEA, and THC chemical mixtures were studied for aerosol size distribution analysis. Two puffing flow rates were applied: 20 mL/s and 40 mL/s (since real-world behavioral data for THC vapers were not available, puffing topography relevant to traditional PG/VG/nicotine-flavor vaping used in our previous study [9] was applied). Count median diameter (CMD) within the 5 nm to 1000 nm size range along with geometric standard deviation (GSD) and total particle number concentration (PNC) up to 10^9^ particles per cc (cubic cm) were measured.

An Electrical Low Pressure Impactor (ELPI+; Dekati Ltd., Kangasala, Finland) that allows particle collection on 14 stages (covering a particle size range from 6 nm to 10,000 nm) was used for selective particle size analysis tests as well as for sample collection for chemical analysis. ELPI+ data and chemical samples were collected at the 430 °F vape pen temperature setting and 40 mL/s flow rate with 40 puffs per sample. VEA, 50%VEA-50%THC, and THC mixtures were used for ELPI+ chemical analysis sampling. Mass median aerodynamic diameter (MMAD) was measured and compared with MMAD calculated using the Hatch–Choate equation [17] from the CMD and GSD data obtained by the DMS500.

### 2.5. Chemical Analysis

Nine aerosol samples—three from vaping of THC, three from vaping of VEA, and three from vaping of 50%/50% THC/VEA—were collected on ELPI+ foils and were extracted so that they could be analyzed for non-targeted chemical analysis. Each set of ELPI+ foils collected from a single vaping session was placed in a separate 30-mL glass jar, to which 15 mL of methylene chloride was added. The jar was sealed and shaken slowly for 15 min at room temperature, and the extract was transferred to a glass bottle. Extraction was repeated twice with 15 mL of methylene chloride each time, combining the three extracts from the same sample to yield 45 mL of extract solution (sample solutions showed large peaks for both THC and VEA, no sample concentration was performed to avoid potential carry-over effects that could be caused by the high boiling temperatures of both THC and VEA) 

Liquid control samples were prepared by adding the appropriate liquids onto clean foils placed inside a 30-mL glass jar, using a mass appropriate to the mass of aerosol collected on the ELPI+ foils from the vaping of the particular liquid. The liquid control samples and aerosol-blank (room-air) control sample were extracted in the same fashion as the test aerosol samples. Aliquots of 15 extract solutions (in some cases, diluted in methylene chloride) were then spiked with a diluted stock solution of the six internal standards (see above) to yield final concentrations of 200 ng/mL for each internal standard prior to non-targeted chemical analysis using two-dimensional gas-chromatography–time-of-flight mass-spectrometry (GC × GC − TOFMS) on a LECO Pegasus 4D instrument (LECO; St Joseph, MI, USA) using the acquisition parameters shown in Table S1. These 15 solutions include 9 from the extractions of aerosol samples, 5 from the extractions of liquid control samples, and 1 from the extraction of the aerosol-blank control sample. All analyte responses (heights) were normalized to the response (height) for the internal standard acenaphthene-d10 (the other five internal standards in the stock solution were used for confirmation). The GC × GC − TOFMS data were evaluated by comparing the chromatographic retention times and the mass spectra of detected signals among the different types of samples.

## 3. Results

### 3.1. Temperature Measurements

**Figure 3:**
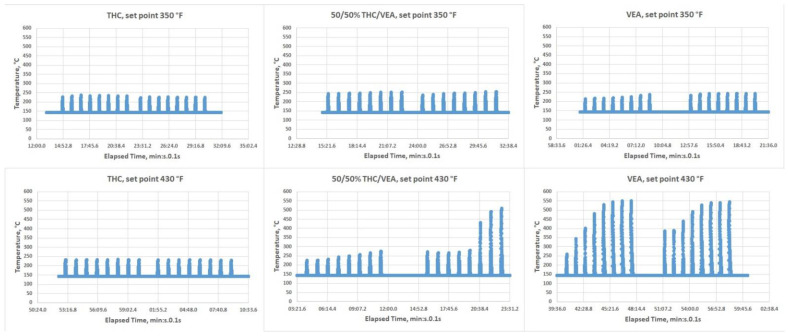
IR temperature measurements (not all data shown, just two eight puffs sessions per test condition).

Figure 3 show the results of the temperature measurements. For THC, regardless of the temperature settings, the temperature normally stayed under 250 °C. For the 50%/50% THC/VEA mixture at the 350 °F set point, the temperature also stayed under 250 °C, but at the 430 °F set point during the second half of the eight puff session, the temperature started to elevate and sometimes reached ~500 °C. For VEA, the temperature measurements at the 350 °F setting stayed under 250 °C, but at the 430 °F set point, temperatures consistently elevated from “puff” to “puff”, quickly reaching a plateau at ~500–550 °C.

### 3.2. Aerosol Size Distribution Measurements

**Figure 4:**
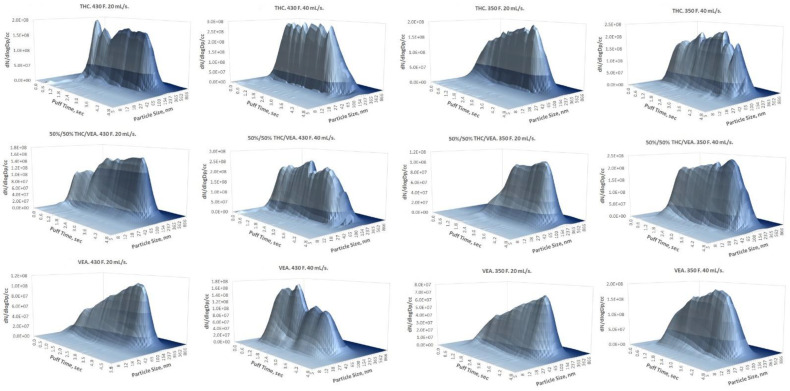
Examples of DMS500 spectrums for all testing conditions. Each plot shows one 5 s puff.

**Figure 5:**
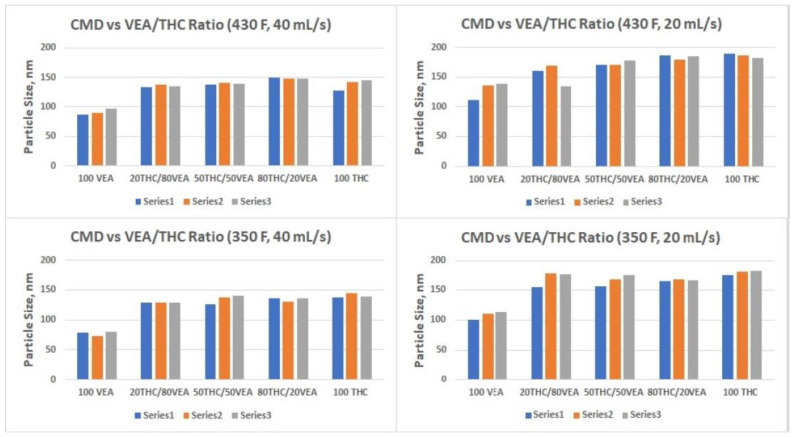
Count Median Diameter vs. Chemical Composition at two temperature settings and two flow rates. Each test was replicated three times. Series number indicates test number.

**Figure 6:**
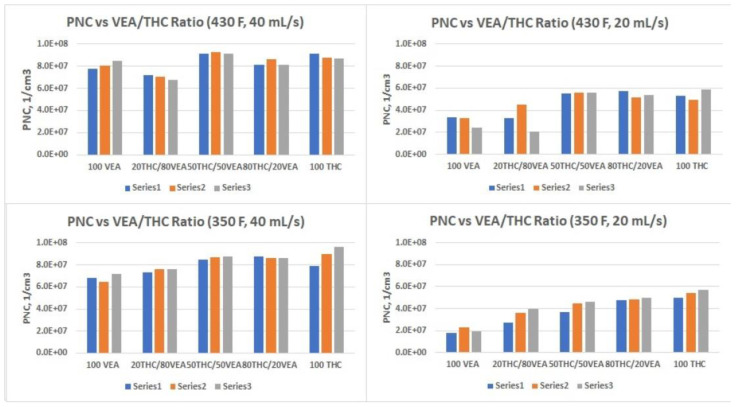
Particle Number Concentration vs. Chemical Composition at two temperature settings and two flow rates. Each test was replicated three times. Series number indicates test number.

Results of the DMS500 measurements are presented in Table 1 and Table 2 and Figure 4, Figure 5 and Figure 6. All particle size distribution spectrums taken for different chemicals mixtures and at both temperature settings showed log-normal distributions (Figure 4). Count median diameter varied from 73 nm to 186 nm with geometrical standard deviations between 1.6 and 1.8. (Table 1). Particle number concentration (Table 2) was within ~2 × 10^7^ to ~1 × 10^8^ particles per cc (cubic cm). The most drastic influence on particle size was observed when chemical composition, as well as flow rate, were changed. VEA aerosol showed the smallest particle size, and an increase of the flow rate further decreased the CMD (to below 100 nm), which was consistent with our previous observations [9]. Changes in heating power had a very slight effect on particle size. 

Particle number concentration was also dependent upon the flow rate, demonstrating a consistent increase as the flow rate increased. At the same time, there was no apparent influence of either chemical content or heating power on the PNC, although some trends could be seen at the 20 mL/s flow rate (Figure 6).

A comparison of MMAD data measured by ELPI+ vs. estimated (using Hatch-Choate equation [17]) MMAD based on CMD and GSD data obtained by DMS500 is presented in Table 3. ELPI+ particle size distribution for THC and 50%/50% THC/VEA chemical mixtures were comparable with DMS500 data (DMS500 vs. ELPI+ data for VEA were presented in our previous study [9]).

### 3.3. Chemical Analysis

For each of the three e-liquids tested (THC, VEA, and 50%/50% THC/VEA), no more than 34 signals were detected for the corresponding aerosol samples, with the numbers being less than twice as large as the numbers of signals detected for the liquid samples themselves. Furthermore, the vaping of each liquid resulted in no more than eight novel signals (i.e., signals that were not detected for liquid samples) being detected for the corresponding aerosol samples (tentative identities assigned to the novel signals by matching mass spectra to the National Institute of Standards and Technology 17 Mass Spectral Library included alkanes, alkenes, alcohols, ketones, and esters). Each of the novel signals detected for any aerosol sample was small in magnitude, having an internal-standard-normalized peak response that was <0.50% as large as that of either THC or VEA for the same sample. Taken together, these results suggest that vaporizing/aerosolizing under the conditions used in the current study led to minimal degradation of either THC or VEA. Comparing the results from the different liquids against one another, it was seen that all detected signals for the aerosol samples of 50%/50% THC/VEA were detected in THC aerosol samples or VEA aerosol samples. Thus, no evidence was found of chemical reactions occurring between THC and VEA during vaporizing/aerosolizing under the conditions used in this investigation.

## 4. Discussion

One of the most concerning observations from the current study as well as from our previous research [9], and independently confirmed by other groups of scientists [8,18], is that the heating temperature of vaping pens that are loaded with viscous e-liquids such as THC/VEA based mixtures may often reach a very high level (500°C–600°C). These high temperatures were observed for both traditional style vape pens equipped with a coil wrapped around a wick [9] and for vape pens specially designed to handle high viscosity mixtures such as dab-type pens with two horizontal coils (see Figure 2 and Figure 3) and ceramic type vertical coil pens [8]. The issue with traditional style vape pens designed for aerosolizing PG-VG based e-liquids, normally equipped with a coil and cotton wick, is most likely associated with the low VEA-THC wick absorption rate (THC-VEA liquid evaporates quicker than the new portion of the liquid is absorbed by the cotton wick and delivered to the coil). The Kind Dream (dab-type) vape pen used for the current study was able to maintain a temperature under 250 °C for all chemical mixtures at the 350 °F temperature setting and for THC at the 430 °F temperature set point. For the 50%/50% THC/VEA mixture at a 430 °F set point, the temperature of the heating coils started to slightly elevate from puff to puff, exceeding the 250 °C level and sometimes reaching ~500 °C. For the VEA at a set point of 430 °F, the temperature of the heating coils consistently rose with each puff reaching a level of ~550 °C. Our visual observations also showed that during heating at the 430 °F set point the VEA liquid not only evaporated but also became less viscous and quickly drained through the holes at the bottom of the tank (those holes were designed for air delivery into the tank). 

These observations indicate that there are certain flaws in the design of vape pens that could affect aerosol generation from THC/VEA mixtures (or other viscous liquids). In order to provide stable and efficient vaporization of chemicals (followed by aerosol formation), the surface of the coil should be in permanent contact with e-liquid. These conditions are certainly easier to achieve for the PG-VG based mixtures due to a high liquid absorption rate by a wick. Loading a high viscosity liquid (THC/VEA) into a traditional vape pen (equipped with a coil wrapped around the cotton wick) may lead to overheating, as we saw in our previous study [9]. For vape pens designed to aerosolize THC/VEA mixtures, a traditional cotton type wick is not normally used [8,18], and chemicals are loaded into the tank equipped either with coils encased in porous ceramic material or coils wrapped around quartz rods. If coils are fully covered with a thick layer of the liquid, then efficient evaporation is not possible (since the majority of the vapor formed at the surface of the coil cannot quickly diffuse through the liquid and form aerosol). Therefore, in order to provide efficient evaporation/aerosolization without overheating the e-liquid, a fine balance has to be achieved when the coils are in contact with chemicals but not fully submerged in a liquid (only a thin layer of chemicals should cover the coils). For high viscosity e-liquids, this balance is hard to achieve and at the high-temperature setting (430 °F) of the dab-type vape pen used for this study, the balance was broken (coils became dry, and the temperature quickly elevated to ~550 °C). Similar results were obtained by another group of researchers [8] who conducted temperature measurements on ceramic type vape pens with vertical coils (50%/50% THC/VEA mixture was used) and found that the average heating temperature varied from 439 °C to 503 °C (maximum temperature reached 569 °C). It also has to be noted that the temperature settings incorporated into the dab-type vape pen by the manufacturer were not accurate: 350 °F and 430 °F should correspond to 177 °C and 221 °C, respectively, and our measurements did not correlate with those setting values. Therefore, a significant risk of overheating exists when vape pens of any type (that were so far studied) are loaded with the high viscosity e-liquids. 

As was already mentioned in the Section 3, a log-normal PSD for all tested aerosol generation conditions was observed as opposed to the bi-modal [19,20,21] or even tri-modal [22] PSD previously reported for e-cigarette aerosol generated out of traditional PG/VG/nicotine/flavor e-liquid mixtures. Multi-modal PSD of traditional e-cigarette aerosol is most likely caused by the simultaneous nucleation of vapors of different groups of compounds with different physical–chemical properties (such as vapor pressure) that leads to the parallel aerosol formation processes generating different particle size modes (usually within the submicron size range). In addition, a larger particle size mode (micron or higher) caused by coagulation was also reported [22]. Unlike PG/VG/nicotine/flavor aerosol, the high molecular weight and low volatility of both THC and VEA [23] resulted in a single-mode PSD in the low submicron (below 200 nm) particle size range. It also has to be noted that due to low volatility, THC/VEA particles are more stable and less sensitive to evaporation than the traditional PG/VG/nicotine/flavor aerosol, therefore while comparing PSD measured for these two types of aerosols, a dilution factor (if applied) should be evaluated since it may enhance the evaporation of the traditional e-cigarette aerosol (particularly affecting particles with a high prevalence of PG).

The smallest aerosol size (nanoparticles below 100 nm CMD) was detected for VEA at the 40 mL/s flow rate (Table 1 and Figure 5), which corresponds with our previous observations [9]. Although it would be beneficial to compare our results with the measurements taken by other researchers for the same compounds, the only data available in the current literature were obtained for VEA using an inertial impaction technique [15] and unfortunately did not allow a direct comparison for several reasons. A different type of vape pen (that was designed to vape PG-VG based mixtures) was used and in order to accurately translate MMAD obtained by the impactor to the CMD obtained by the differential (electrical) mobility technique using the Hatch–Choate equation [17], a log-normal distribution is required. Based on a very high GSD (2.35) measured for the VEA aerosol using an inertial impactor [15], it is hard to assume the log-normality of the PSD. When applying the Hatch–Choate equation using the published data (MMAD = 610 nm, GSD = 2.35) [15], the estimated CMD would be ~69 nm, but that probably cannot be considered a valid MMAD to CMD conversion due to the reasons explained above. Our data showed that a high concentration of nanoparticles (below 100 nm CMD) could be generated under certain conditions, such as high flow rate and the prevalence of VEA in the e-liquid used, with the dab-type vape pen used for the study. The hydrophobic nature of those nanoparticles may prevent their substantial growth (due to lack of water absorption) while passing through the human respiratory system, hence allowing penetration into the deep regions of the lungs and can potentially cause a variety of toxic effects [24,25,26].

Another goal of this study was to define how chemical content (THC, VEA, and their mixture) affected possible chemical transformations, whether heating could lead to thermal degradation of either THC or VEA (or both), and if there is a chemical interaction between the heated THC and VEA. For the current study, chemical transformations of THC and VEA were not detected or detected at a minimal level. As temperature measurements showed, THC aerosol generation occurred at temperatures within the 250 °C level, and for the 50%/50% THC/VEA mixture, the heating temperature only slightly exceeded 250 °C and only occasionally rose to ~500 °C. Therefore, for the type of vape pen used, neither THC degradation nor THC-VEA chemical interaction were detected. With regard to VEA heating, minimal thermal degradation was observed in the current study as opposed to much higher degradation levels found in our previous research [9]. Despite this, similar high heating temperatures (500–600 °C) were measured in both studies. This difference in VEA chemical conversion can most likely be attributed to the different types of vape pens used in these two studies. It is suspected that in the previous work, the cotton wick allowed a longer exposure of the VEA to the high temperature, hence enhanced thermal degradation of VEA was seen (although liquid quickly evaporated from the surface of the coil and was not able to provide a cooling effect, VEA could still reside in the wick exposed to high temperature for the entire duration of the puff). Dab-type vape pens used in the current study utilized titanium coils wrapped around quartz rods (as opposed to a cotton wick used in the previous study); therefore, heated VEA quickly flowed down the coils providing less time for VEA contact with the heated coil. Additionally, the Joyetech eVic vape pen used in the previous study was supplied with a nichrome coil, hence the differences in catalytic properties of nichrome vs. titanium may also play a role. 

Ideally, to make a comparative assessment of the chemical transformations as a function of chemical content, heating conditions (such as temperature and duration of the heating and the type of coil/wick) should be the same for all chemicals and their mixtures tested. Apparently, commercially available vape pens are not well suited for that type of comparison. Dab-type vape pens are designed for wax-type materials (such as THC) and are not very suitable for oil-type e-liquids (such as VEA and THC/VEA mixtures with a significant amount of VEA), whereas vape pens designed for liquid oils are not suitable for wax. With regard to possible regulatory implications for vape pen design and e-liquids, it is important to consider not only heating power (and temperature), type of the coil/wick material, and chemical content of e-liquids, but also the types of e-liquids that are suitable for the certain types of vape pens. The design properties of the vape pen in combination with an improper type of e-liquid could lead to quick-drying and overheating of the coil. As our measurements showed, temperature controls implemented on some commercially available vape pens could also be compromised.

## 5. Limitations

As we already mentioned, since the entire range of THC/VEA mixtures (VEA, 20%/80% THC/VEA, 50%/50% THC/VEA, 80%/20% THC/VEA, and THC) was planned to be studied using the same aerosol generation conditions (the same vape pen, heating power settings, and puffing topography) we had to restrict our choice to a dab-type vape pen that could be used with all these mixtures including both solid (wax) and liquid states. As we found, the use of dab-type vape pens leads to a reduced heating time of the VEA (and VEA/THC mixtures); therefore, vape pens designed for oil-type e-liquids should be used to study the possible thermal degradation of liquid chemicals that could occur in real-world use.

While the GC × GC − TOFMS results did not provide evidence of chemical reactions between THC and VEA during vaporizing/aerosolizing, it is possible that such reactions may occur but were not detected by the methods employed in this study. For example, reaction products that are highly volatile were not collected on the ELPI+ foils and thus were absent from the extracts analyzed by GC × GC − TOFMS. Other possible reasons that reaction products—if they were present—were not detected include products that are not effectively extracted by methylene chloride (an organic solvent of moderate polarity), products that are not amenable to gas chromatographic analysis due to having low vapor pressures, and products that are present at very low concentrations. Future research to investigate the possibility of reactions between THC and VEA during vaping could use a more extensive suite of techniques for sample collection, extraction, and analysis to address some of these limitations, but method sensitivity is always a constraint with regard to the possible inability to detect products at very low concentrations.

## Figures and Tables

**Table 1:** Summary of the DMS500 Data. Each data point shows average count median diameter (along with geometric standard deviation) across an eight puff session per testing condition (such as temperature set point, chemical content, and puffing flow rate). Each test was replicated three times.

Temperature Set Point and Test#	VEA 20 mL/s CMD (GSD) nm	VEA 40 mL/s CMD (GSD) nm	20THC/80VEA 20 mL/s CMD (GSD) nm	20THC/80VEA 40 mL/s CMD (GSD) nm	50THC/50VEA 20 mL/s CMD (GSD) nm	50THC/50VEA 40 mL/s CMD (GSD) nm	80THC/20VEA 20 mL/s CMD (GSD) nm	80THC/20VEA 40 mL/s CMD (GSD) nm	THC 20 mL/s CMD (GSD) nm	THC 40 mL/s CMD (GSD) nm
430 °F Test-1	111 (1.7)	87 (1.7)	160 (1.7)	133 (1.6)	171 (1.6)	137 (1.7)	186 (1.6)	149 (1.7)	190 (1.6)	128 (1.6)
430 °F Test-2	136 (1.7)	89 (1.7)	169 (1.6)	138 (1.6)	171 (1.6)	141 (1.6)	179 (1.6)	148 (1.6)	187 (1.7)	142 (1.6)
430 °F Test-3	139 (1.7)	97 (1.7)	135 (1.8)	134 (1.6)	177 (1.6)	140 (1.6)	185 (1.6)	148 (1.7)	183 (1.6)	145 (1.6)
350 °F Test-1	101 (1.8)	79 (1.8)	154 (1.7)	128 (1.6)	155 (1.7)	126 (1.7)	165 (1.7)	136 (1.7)	175 (1.6)	137 (1.7)
350 °F Test-2	111 (1.8)	73 (1.8)	177 (1.6)	128 (1.6)	167 (1.7)	137 (1.7)	168 (1.7)	130 (1.7)	180 (1.6)	145 (1.6)
350 °F Test-3	114 (1.8)	80 (1.7)	176 (1.6)	129 (1.6)	175 (1.7)	141 (1.7)	166 (1.7)	137 (1.7)	181 (1.6)	140 (1.6)

**Table 2:** Summary of the DMS500 Data. Each data point shows the average particle number concentration across an eight puff session per testing condition (such as temperature set point, chemical content, and puffing flow rate). Each test was replicated three times.

Temperature Set Point and Test#	VEA 20 mL/s PNC N/cc	VEA 40 mL/s PNC N/cc	20THC/80VEA 20 mL/s PNC N/cc	20THC/80VEA 40 mL/s PNC N/cc	50THC/50VEA 20 mL/s PNC N/cc	50THC/50VEA 40 mL/s PNC/cc	80THC/20VEA 20 mL/s PNC N/cc	80THC/20VEA 40 mL/s PNC N/cc	THC 20 mL/s PNC N/cc	THC 40 mL/s PNC N/cc
430 °F Test-1	3.3E+07	7.8E+07	3.3E+07	7.2E+07	5.5E+07	9.1E+07	5.7E+07	8.1E+07	5.3E+07	9.1E+07
430 °F Test-2	3.3E+07	8.1E+07	4.5E+07	7.0E+07	5.6E+07	9.3E+07	5.2E+07	8.6E+07	4.9E+07	8.8E+07
430 °F Test-3	2.5E+07	8.5E+07	2.1E+07	6.8E+07	5.6E+07	9.1E+07	5.4E+07	8.1E+07	5.9E+07	8.7E+07
350 °F Test-1	1.8E+07	6.8E+07	2.7E+07	7.3E+07	3.7E+07	8.5E+07	4.8E+07	8.8E+07	5.0E+07	7.9E+07
350 °F Test-2	2.3E+07	6.5E+07	3.6E+07	7.6E+07	4.5E+07	8.7E+07	4.8E+07	8.6E+07	5.4E+07	9.0E+07
350 °F Test-3	2.0E+07	7.2E+07	4.0E+07	7.6E+07	4.6E+07	8.8E+07	4.9E+07	8.6E+07	5.7E+07	9.7E+07

**Table 3:** MMAD measured by ELPI+ vs. MMAD estimated using Hatch–Choate equation from CMD and GSD measured by DMS500. Temperature set point 430 °F, flow rate 40 mL/s. Each test was replicated three times.

Instrument	ELPI Measured MMAD, nm	DMS500 Estimated MMAD, nm	ELPI Measured MMAD, nm	DMS500 Estimated MMAD, nm
Chemical Content	50THC/50VEA	50THC/50VEA	THC	THC
Test-1	238	294	319	244
Test-2	221	291	310	301
Test-3	246	278	345	305

## Data Availability

Not applicable.

## Supplementary Materials

The following supporting information can be downloaded at: https://www.mdpi.com/article/10.3390/toxics10020088/s1. Table S1: Acquisition parameters for non-targeted chemical analysis by two-dimensional gas-chromatography–time-of-flight mass-spectrometry (GC × GC − TOFMS).
