# Supplementary material for: Analysis of the Aerosol Generated from Tetrahydrocannabinol, Vitamin E Acetate, and Their Mixtures
Source: Toxics. 2022 Feb 15;10(2):88. doi: 10.3390/toxics10020088 (PMC8878975; doi:10.3390/toxics10020088)
Supplement: Supplementary file 1 [file toxics-10-00088-s001.zip › toxics-1586873-supplementary.pdf]

toxics

MDPI

Supplementary Materials: Analysis of the Aerosol Generated from Tetrahydrocannabinol, Vitamin E Acetate, and Their Mixtures

Vladimir B. Mikheev and Alexander Ivanov

Table S1. Acquisition parameters for non-targeted chemical analysis by two-dimensional gas-chromatography–time-of-flight mass-spectrometry (GC × GC-TOFMS).

GC×GC-TOFMS system	LECO Pegasus 4D
GC 1st-dimension column	100% dimethylpolysiloxane, 30 m, 0.25 mm i.d., 1 μm d_f
GC 2nd-dimension column	50% phenyl polysilphenylene-siloxane 1 m, 0.1 mm i.d., 0.1 μm d_f
GC carrier gas	Helium
GC carrier gas flow rate	Constant flow, 1.3 mL/min
GC inlet temperature	250 °C
GC injection volume	1 μL splitless
GC 1st-dimension column oven program	45 °C (1.5 min hold)<br>45 °C–100 °C @ 20 °C/min<br>100 °C–270 °C @ 3 °C/min; hold 1 min<br>270 °C–320 °C @ 20 °C/min; hold 26 min
GC 2nd-dimension column oven program	80 °C (1.5 min hold)<br>80 °C–275 °C @ 3 °C/min<br>275°C – 330 °C @ 20 °C/min; hold 21 min
GC modulator temperature offset from first oven	+20 °C
GC modulation time	3 seconds
Transfer line temperature	290 °C
MS source temperature	200 °C
MS detector voltage	Tune voltage + 200 V
MS acquisition masses	35–600 amu
MS data acquisition rate	100 spectra per second
MS resolving power	Unit mass resolution
